# Genome Sequence of *Anoxybacillus thermarum* AF/04^T^, Isolated from the Euganean Hot Springs in Abano Terme, Italy

**DOI:** 10.1128/genomeA.00490-15

**Published:** 2015-05-21

**Authors:** Annarita Poli, Barbara Nicolaus, Kok-Gan Chan, Ummirul Mukminin Kahar, Chia Sing Chan, Kian Mau Goh

**Affiliations:** a Institute of Biomolecular Chemistry (ICB), Consiglio Nazionale delle Ricerche (CNR), Pozzuoli, Naples, Italy; b Faculty of Science, Division of Genetics and Molecular Biology, Institute of Biological Sciences, University of Malaya, Kuala Lumpur, Malaysia; c Faculty of Biosciences and Medical Engineering, Universiti Teknologi Malaysia, Skudai, Johor, Malaysia

## Abstract

*Anoxybacillus thermarum* AF/04^T^ was isolated from the Euganean hot springs in Abano Terme, Italy. The present work reports a high-quality draft genome sequence of strain AF/04^T^. This work also provides useful insights into glycoside hydrolases, glycoside transferases, and sugar transporters that may be involved in cellular carbohydrate metabolism.

## GENOME ANNOUNCEMENT

*Anoxybacillus* spp. are Gram-positive alkalothermophilic bacteria that thrive in a variety of habitats (1). *Anoxybacillus* spp. have garnered interest for many biotechnological applications, such as in the starch and lignocellulose degradation industry, bioremediation processes, and as a candidate for a thermophilic microbial cell factory (1, 2).

*Anoxybacillus thermarum* AF/04^T^ (= DSM 17141^T^ = ATCC BAA 1156^T^) was isolated from thermal mud at the Euganean hot springs in Abano Terme, Italy (3). Strain AF/04^T^ is an aerobic and thermophilic bacterium with optimal growth at 65°C and pH 7.2 (3). Carbon source utilization tests showed that strain AF/04^T^ is able to grow only in the presence of maltose, trehalose, and sodium acetate (3), while most *Anoxybacillus* spp. utilize a wide range of carbon sources (1). These differences in carbon source utilization led us to sequence the genome of strain AF/04^T^. The high-quality annotated draft genome of strain AF/04^T^ might provide insights essential to understanding carbohydrate metabolism by its cells and other biochemical networks of the strain. The genomic study may also shed new light on its potential usability in industrial processes.

The genomic DNA of strain AF/04^T^ was extracted using a Qiagen DNeasy blood and tissue kit (Qiagen, Hilden, Germany), according to the manufacturer’s protocol. The paired-end library was prepared using the standard protocol of the Nextera DNA sample preparation kit (Illumina, San Diego, CA, USA). Sequencing was performed using the Illumina MiSeq sequencing platform with 300-bp paired-end reads. The adapter sequences, low-quality regions, and reads were filtered out using Trimmomatic (4), Scythe (UC Davis Bioinformatics Core, Davis, CA, USA), and SGA (5), respectively. Next, the reads were subjected to *de novo* genome assembly using IDBA-UD 1.0.9 (6) to a coverage of 270×. The high-quality draft genome is 2,736,908 bp in size, with a G+C content of 42%. The total coding region is 2,390,322 bp, with a total of 2,922 genes, of which 2,828 are protein coding. A total of 11 rRNA genes, 84 tRNA genes, and 1 transfer-messenger RNA (tmRNA) gene were predicted.

The AF/04^T^ annotated genome revealed various glycoside hydrolase (GH) and glycoside transferase (GT) enzymes that are possibly involved in cellular carbohydrate metabolism. The GHs (i.e., α-amylase, β-glucosidase, trehalose-6-phosphate hydrolase, 1,4-α-glucan branching enzyme, oligo-1,6-glucosidase, α-glucosidase, and sucrase-6-phosphate hydrolase) and GTs (i.e., maltodextrin phosphorylase and glycogen synthase) may be required for cellular starch and sucrose metabolism. Some of the aforementioned GHs (i.e., oligo-1,6-glucosidase and α-glucosidase) may also be involved in cellular galactose metabolism. Genes encoding several putative sugar transporters, such as ATP-binding cassette (ABC) and phosphotransferase system (PTS) transporters, were also found in the genome, suggesting that these transporters may facilitate sugar uptake by the cells from the environment. Thus, the aforementioned GHs, GTs, and sugar transporters are worthy of further examination to gain a possible explanation for the limited carbon uptake behavior of strain AF/04^T^.

### Nucleotide sequence accession numbers.

This whole-genome shotgun project has been deposited in DDBJ/EMBL/GenBank under the accession no. JXTH00000000. The version described in this paper is the first version, JXTH01000000.
